# A Wireless Passive Pressure-Sensing Method for Cryogenic Applications Using Magnetoresistors

**DOI:** 10.3390/s24030717

**Published:** 2024-01-23

**Authors:** Ziqi Zhao, Michitaka Yamamoto, Seiichi Takamatsu, Toshihiro Itoh

**Affiliations:** Department of Precision Engineering, Graduate School of Engineering, The University of Tokyo, Tokyo 113-8656, Japan

**Keywords:** pressure sensing, cryogenic, magnetoresistor, backscattering, wireless, passive

## Abstract

In this study, we developed a novel wireless, passive pressure-sensing method functional at cryogenic temperatures (−196 °C). The currently used pressure sensors are inconvenient and complicated in cryogenic environments for their weak low-temperature tolerances and long wires for power supply and data transmission. We propose a novel pressure-sensing method for cryogenic applications by only using low-temperature-tolerant passive devices. By innovatively integrating a magnetoresistor (MR) on a backscattering antenna, the pressure inside a cryogenic environment is transferred to a wirelessly obtainable return loss. Wireless passive measurement is thus achieved using a backscattering method. In the measurement, the pressure causes a relative displacement between the MR and a magnet. The MR’s resistance changes with the varied magnetic field, thus modulating the antenna’s return loss. The experimental results indicate that our fabricated sensor successfully identified different pressures, with high sensitivities of 4.3 dB/MPa at room temperature (24 °C) and 1.3 dB/MPa at cryogenic temperature (−196 °C). Additionally, our method allows for simultaneous wireless readings of multi sensors via a single reading device by separating the frequency band of each sensor. Our method performs low-cost, simple, robust, passive, and wireless pressure measurement at −196 °C; thus, it is desirable for cryogenic applications.

## 1. Introduction

Pressure sensing is a crucial issue in a wide range of industrial applications such as fault detection [1,2] and process control [3,4,5]. Cracking and leaking, resulting in catastrophic failures, can be prevented through the monitoring and analysis of pressure. As technology advances, pressure sensing is becoming a key issue in the cryogenic community. Key examples include the monitoring of aerospace equipment such as a spacecraft [6,7] and the monitoring of automated production lines such as a cryogenic gas recovery plant [8,9,10]. Pressure monitoring in a spacecraft can help to indicate the equipment condition and maintain a stable operation over a long period. Pressure monitoring in cryogenic chambers or pipelines can help to detect potential cracks [1,10,11] and extract the desired cryogenic fuel from the exhaust gases [8,9].

In these cryogenic applications, pressure monitoring has three main challenges. First, most sensors are not functional at cryogenic temperatures (e.g., the temperature of liquified natural gas is −161 °C). At such a low temperature, most circuits that include microcontrollers and wireless ICs cannot survive. Second, the currently used cryogenic pressure sensors are wired sensors because multi-layer metal shields are used for thermal insulation. The metal shields block the transmission and reception of wireless signals. For large sensor arrays which are distributed in a wide area, the wirings for each sensor are rather complicated and costly. Third, the maintenance is difficult because sensors that are embedded in cryogenic environments are physically inaccessible. To overcome the challenges, a wireless passive pressure sensor that is functional at cryogenic temperatures is desirable.

Backscattering is a promising method to achieve wireless passive measurement at cryogenic temperatures due to its robust characteristic in extreme conditions and the simple structure of the required sensors [6,12,13]. In recent years, diverse wireless passive sensors have been developed through the backscattering method, measuring pressure [14,15,16,17,18], strain [19,20], vibration [21], temperature [22,23], etc. Backscattering pressure sensors mainly include four types: inductive–capacitive (LC) resonance sensors, microwave scattering resonance sensors, surface acoustic wave (SAW) sensors, and radio frequency identification (RFID) sensors. As for LC resonance sensors, Jia et al. used the pressure-induced deformation of an MgO-sensitive membrane to modulate the sensor’s center frequency. Wireless passive pressure measurement is achieved at 900 °C [14]. In their research, the reading device is placed only several millimeters away from the sensor. In cryogenic applications, such a read range is not enough to read the embedded sensors. As for microwave scattering resonance sensors, Su et al. used a slot antenna to achieve pressure measurement [15]. The applied pressure changes the structure of the slot antenna, thus modulating the antenna’s center frequency. The sensor works in the microwave band and is expected to have a satisfying read range, but the shift in the sensor’s center frequency negatively affects the implementation of large sensor arrays. To decouple the monitored signal and differentiate the backscattered signal from each sensor, sensors need to work in separate frequency bands. The sensor’s large frequency shift indicates that each sensor occupies a broad bandwidth, which negatively influences the maximum number of sensors in a sensor array. As for SAW sensors, Tang et al. used a bidirectional reflective delay line to transfer the applied pressure to the phase shift of the SAW signal [16]. Since the maximum operating frequency of SAW devices is several gigahertz, the maximum number of sensors in a sensor array is also limited. As for RFID sensors, Feng et al. developed a self-tuning RFID tag to achieve a robust monitoring of liquid pressure. In their research, an RFID chip is necessary for wireless communication, which is not functional for long-term monitoring at cryogenic temperatures [17]. Although chipless RFID sensors are independent of RFID chips [18], they are similar to microwave scattering resonance sensors and have limitations in the implementation of large sensor arrays. Additionally, no research has been undertaken to investigate the cryogenic performance of backscattering pressure sensors.

In our previous work [21], a wireless passive vibration sensing method is proposed for cryogenic applications. By integrating a magnetoresistor (MR) on a backscattering antenna, the vibration induces a relative displacement between the MR and a magnet, thus modulating the wireless characteristics of the antenna. In this paper, we found that the sensing principle in our previous work can also perform pressure sensing by transforming the pressure to a relative displacement between the MR and the magnet. Furthermore, the antenna design and the load design are optimized to enhance the sensor’s linearity and sensitivity. Additionally, the simultaneous wireless reading of multi sensors is achieved via a single reading device.

Herein, we present a novel wireless passive pressure sensor that functions at cryogenic temperatures, distinguished from previous works by achieving the following four goals simultaneously: (1) develop a novel pressure-sensing mechanism which operates in a wide temperature range, including cryogenic temperatures; (2) enable wireless communication with a good read range to obtain the data from a physically inaccessible sensor; (3) design a passive sensor to reduce maintenance; and (4) achieve simultaneous monitoring with multi sensors with only one reading device.

## 2. Materials and Methods

### 2.1. The Proposed Wireless Passive Pressure-Sensing Method

**Figure 1:**
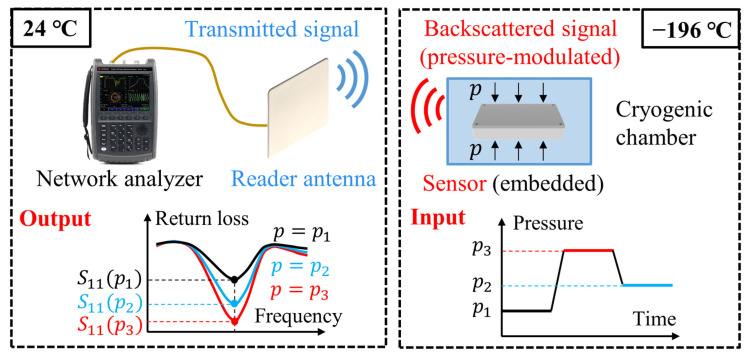
A schematic of the proposed wireless, passive pressure-sensing method for cryogenic applications. The sensor’s return loss is wirelessly monitored via the backscattering method, which is then used to derive the applied pressure.

**Figure 2:**
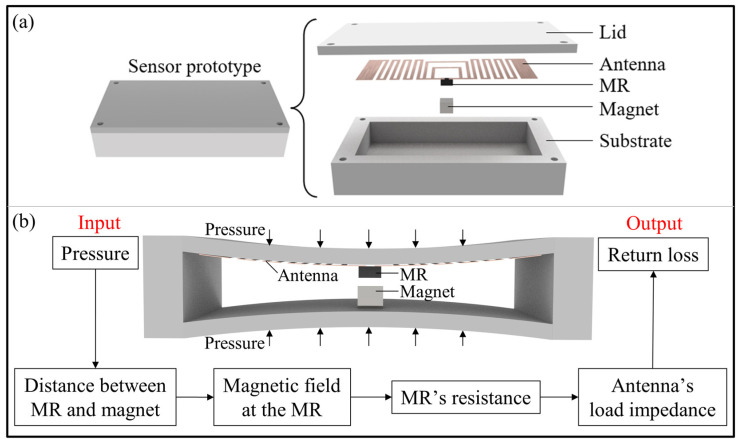
The sensing mechanism and a prototype of the designed wireless passive pressure sensor. (**a**) The structure of the sensor prototype. (**b**) The passive pressure-sensing mechanism. The pressure causes a deformation in the sensor’s package and adjusts the distance between the magnetoresistor (MR) and the magnet. The magnetic field intensity at the position of the MR changes accordingly, leading to a variation in the MR’s resistance. Since the MR is part of the antenna’s load, the antenna’s impedance is mismatched to its load impedance. Thus, the antenna’s return loss changes with the pressure.

In the proposed wireless, passive pressure-sensing method, the sensor is embedded in the monitored cryogenic environment, as shown in Figure 1. To obtain the data from such a physically inaccessible sensor, the backscattering method is used. The backscattering system, including a reader antenna and a network analyzer, works outside the cryogenic environment. In the measurement, the sensor passively receives the wireless signal from the reader antenna and then passively reflects a backscattered signal to the reader antenna. The backscattered signal is monitored and processed in the network analyzer to obtain the return loss of the sensor. The return loss is modulated by the applied pressure (which will be explained later in Figure 2). As a result, the return loss of an embedded sensor can be monitored wirelessly, which can be used to derive the applied pressure.

To passively modulate the sensor’s return loss with the applied pressure, a novel pressure-sensing mechanism is proposed. The main components include an antenna, a magnetoresistor (MR), and a magnet, as shown in Figure 2a. All employed components are passive devices and tolerant to extremely low temperatures; thus, the proposed method is functional at cryogenic temperatures. In the designed sensor, the antenna is fixed on the inner surface of the lid, and the magnet is fixed on the inner surface of the substrate. The MR is connected to the antenna, performing as a part of the antenna’s load. As shown in Figure 2b, the applied pressure on the sensor causes a deformation in the sensor’s package due to the sensor’s hollow internal structure. Due to such a pressure-induced deformation, the distance between the MR and the magnet becomes closer. As a result, the magnetic field intensity at the position of the MR changes accordingly. The variation in the magnetic field intensity affects the MR’s resistance due to the magnetoresistance effect. Since the MR is a part of the antenna’s load, the load impedance of the antenna also changes. The impedance matching between the antenna and the load is tuned accordingly, thus modulating the sensor’s return loss. In this way, the applied pressure on the sensor can passively modulate the sensor’s return loss.

The developed pressure sensor is functional at cryogenic temperatures because all employed components are passive and low-temperature-tolerant. The antenna is composed of a metal film and a plastic substrate, the core structure of the MR is multi-layers of metal film, and the magnet is made of a metal material. Thus, the designed sensor is applicable to cryogenic applications.

In summary, the pressure applied on the designed sensor passively modulates the sensor’s return loss. The return loss is then wirelessly monitored in a network analyzer through a backscattering system. Since all components of the sensor are functional at cryogenic temperatures, the pressure inside a cryogenic environment can be wirelessly monitored by the passive sensor.

### 2.2. Theoretical Explanation of the Sensing Mechanism

As described in Section 2.1, when a uniform pressure (p) is applied on the sensor, the sensor’s lid and substrate will be deformed. Due to such a pressure-induced deformation, the distance between the MR and the magnet becomes closer. As a result, the magnetic field intensity at the position of the MR changes accordingly.

According to the MR’s characteristic, such a variation in the magnetic field intensity will lead to a variation in the MR’s resistance, as shown in Equation (1).

(1) $$Z_{MR}=R_{0}\left[1+\alpha \left(B_{MR}\right)\right]$$

In Equation (1), $R_{0}$ is the MR’s resistance when the magnetic field intensity is 0, $B_{MR}$ is the magnetic field intensity along the MR’s sensitive direction, and $\alpha \left(B_{MR}\right)$ is the percentage of the MR’s resistance variation.

For a better performance of the sensor’s antenna, a capacitor could be used for impedance matching. If the MR is connected in series with the capacitor, the load of the sensor’s antenna will include the capacitor and the MR; the equivalent load impedance, $Z_{L},$ can be calculated in Equation (2).

(2) $$Z_{L}=Z_{C}+Z_{MR}$$

In Equation (2), $Z_{C},Z_{MR}$ are the impedance of the capacitor and the MR, respectively. The return loss of the sensor’s antenna is expressed in Equation (3).

(3) $$S_{11}=20\times \mathrm{lg}\left|\frac{Z_{L}-Z_{A}^{*}}{Z_{L}+Z_{A}}\right|$$

In Equation (3), $S_{11}$ is the return loss of the sensor’s antenna. $Z_{A}$ is the impedance of the sensor’s antenna; $Z_{A}^{*}$ is the conjugate of $Z_{A}$.

In summary, there is a strong correlation between the return loss of the sensor’s antenna and the monitored pressure. The feasibility of the proposed pressure-sensing method is verified theoretically. 

### 2.3. The Design and Fabrication of the Sensor Prototype

#### 2.3.1. Antenna Design

**Figure 3:**
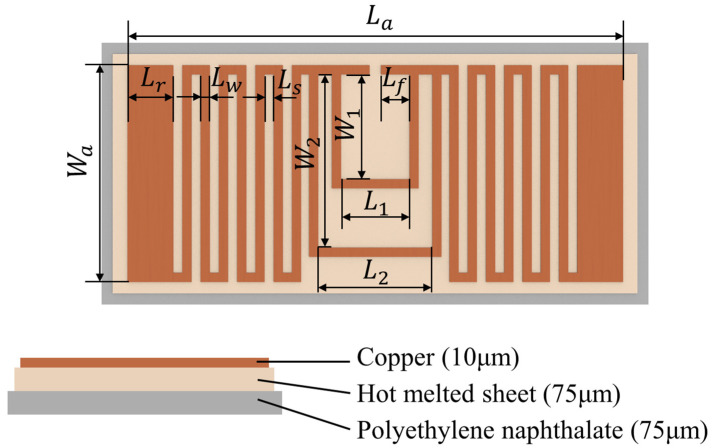
The dimension and structure of the designed antenna.

In the designed sensor prototype, the center frequency of the antenna is designed in the UHF bandwidth. This is because antennas in the UHF bandwidth can achieve a good read range with both a small size and a low cost. The sensor’s antenna is composed of a copper conductor layer, a polyethylene naphthalate (PEN) substrate layer, and a hot melted sheet (HMS) layer for bonding the copper layer and the PEN substrate layer together. The dimension and structure of the designed antenna are shown in Figure 3 and Table 1.

In the fabrication of the antenna, firstly, the three layers in Figure 3 are bonded together via hot pressing. The hot melted sheet performs as an adhesive to bond the copper layer and the PEN substrate layer together. Secondly, the copper layer of the antenna is fabricated into the designed pattern with a cutting plotter machine (FCX4000-50ES, Graphtec, Kanagawa, Japan). Finally, the edge of the antenna is cut off for the assembly of the sensor.

#### 2.3.2. Load Design

As introduced in the sensor’s working principle (Figure 2), an MR is used to sense the pressure-induced magnetic field variation and then modulate the antenna’s return loss. To achieve a high sensitivity, the selected MR should have a high MR ratio. Additionally, the impedance of the load should match the impedance of the designed antenna.

**Figure 4:**
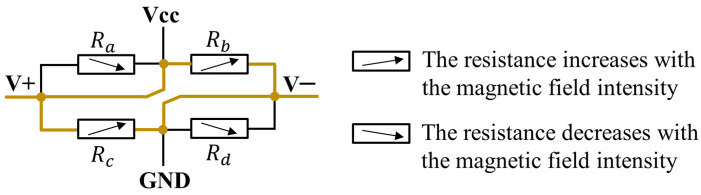
The equivalent internal circuit of a passively worked TMR.

As for the MR, tunnel magnetoresistors (TMRs) are the best choices for their highest sensitivity and smallest size among all kinds of MRs [24]. In the selected TMR (TMR2104LS, MDT), four MRs form a Wheatstone bridge, as shown in Figure 4. In the designed sensing principle, the TMR works passively. The equivalent resistance between port V+ and port V− changes with the magnetic field intensity along the TMR’s sensing direction. To ensure a maximum variation in the TMR resistance, resistor R_a_ (between V+ and Vcc), as well as resistor R_d_ (between V− and GND) are shorted. This is because, as the magnetic field varies, the responses of the shorted two resistors (R_a_ and R_d_) are opposite to the responses of the remaining two resistors (R_b_ and R_c_).

**Figure 5:**
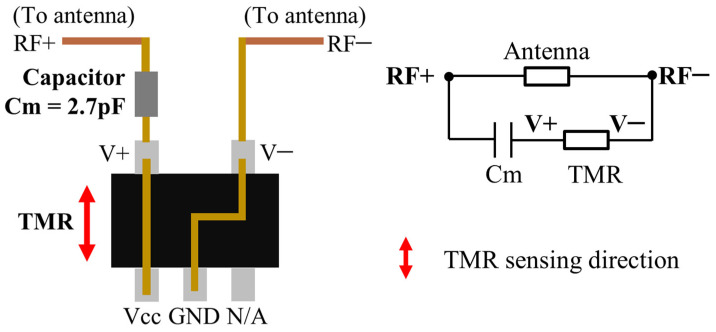
The load design of the designed wireless passive pressure sensor.

To match the impedance between the antenna and the load, the TMR is in a series connection with a 2.7 pF capacitor, as shown in Figure 5. In the TMR, port V+ is connected to port Vcc, and port V− is connected to port GND, as analyzed in Figure 4. To integrate the load on the antenna, the 2.7 pF capacitor (Cm) is connected to port V+ of the TMR and one feed point of the antenna (RF+). The other feed point of the antenna (RF−) is connected to port V− of the TMR.

#### 2.3.3. The Package of the Sensor

In the cryogenic community, PTFE was widely used for its chemical stability and versatility in a wide range of temperatures [22,25]. However, PTFE structures are hard to be bonded because PTFE material has a low surface energy. This brings challenges to fabricating watertight or airtight sensors. Additionally, PTFE is processed via subtractive manufacturing. In massive production, a large quantity of materials is removed and wasted.

To avoid these problems, the package of the sensor is made of PETG material. PETG is also applicable to cryogenic environments and is chemical-resistant [26]. Additionally, it can be easily fabricated through 3D-printing and can be easily bonded with diverse cryogenic adhesives, which make it possible for low-cost, massive production.

#### 2.3.4. The Assembly of the Sensor

The assembly of the sensor includes four steps. First, the load is soldered onto the antenna. Second, the load-integrated antenna is fixed on the inner surface of the lid using a cryogenic adhesive. Third, the magnet is fixed on the inner surface of the substrate using a cryogenic adhesive. Fourth, the lid and the substrate are bonded together with a cryogenic adhesive. The overall size of the designed sensor is 57.0 mm × 30.0 mm × 10.0 mm. The total cost of the designed sensor is about USD 2.38, as shown in Table 2.

**Figure 6:**
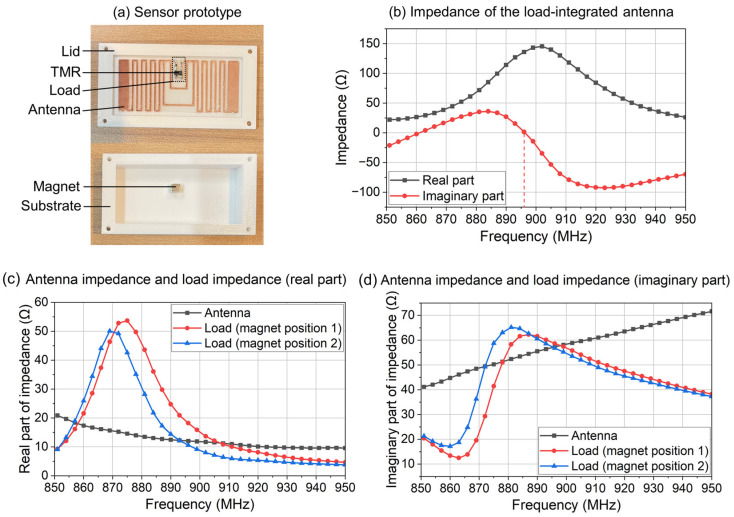
(**a**) The fabricated sensor prototype. (**b**) The impedance of the load-integrated antenna. (**c**) The real part of the antenna impedance and the load impedance. The load impedance is measured at two different magnet positions. (**d**) The imaginary part of the antenna impedance and the load impedance. The load impedance is measured at two different magnet positions.

The fabricated sensor prototype is shown in Figure 6a, and the impedance of the load-integrated antenna is measured as Figure 6b. The antenna impedance and the load impedance are also measured separately, as shown in Figure 6c,d. In Figure 6c,d, the load impedance is measured at two different magnet positions.

Figure 6b shows that impedance matching is achieved at around 896 MHz (marked by the dotted line). As the magnet moves from position 1 to position 2, the real part of the load impedance at 896 MHz decreases by 41.8% (from 18.2 Ω to 10.6 Ω), and the imaginary part of the load impedance at 896 MHz decreases by 3.1% (from 58.6 Ω to 56.8 Ω). For the impedance of the designed load, the variation in the real part is much more obvious than the variation in the imaginary part. This indicates that the center frequency of the sensor is stable during the pressure measurement.

### 2.4. The Test Bench

**Figure 7:**
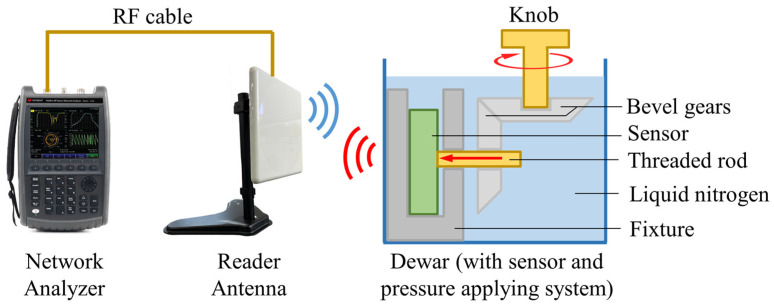
The test bench of the pressure-sensing experiment. The output power of the reader antenna is 5 dBm. The distance between the reader antenna and the sensor is about 35 cm. For simplification, the structure of the pressure-applying system (structures inside the dewar) is not detailed.

To prove that the proposed pressure-sensing method and the fabricated sensor prototype are functional for wireless passive measurement at cryogenic temperatures, a pressure measurement experiment is conducted. The test bench is composed of a 9 dBi gain reader antenna (VAL-262006, Vulcan RFID, Birmingham, AL, USA), a network analyzer (N9923A, Agilent Technologies, Santa Clara, CA, USA), a dewar (175 mL, Spearlab, San Francisco, CA, USA), a pressure-applying system, and the sensor prototype, as shown in Figure 7.

**Figure 8:**
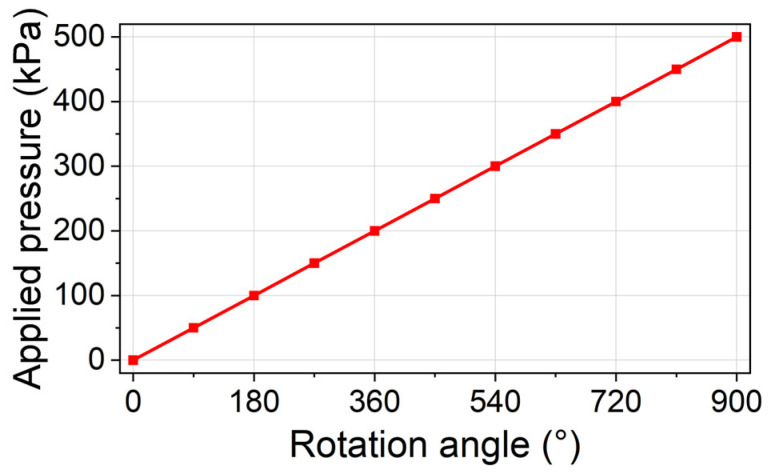
The calibrated relationship between the rotation angle of the knob and the applied pressure.

In the experiment, the sensor is submerged in liquid nitrogen (−196 °C). A pressure-applying system is designed for applying an adjustable pressure on the sensor. The pressure-applying system is composed of a knob, a pair of bevel gears, a threaded rod, and a fixture. The threaded rod is connected to the bevel gear through key connection, and it is connected to the fixture through threaded connection. As a result, the horizontal rotation of the knob will be transformed to a vertical rotation of the threaded rod via the bevel gears. Such a vertical rotation will lead to a horizontal displacement of the threaded rod, because of the threaded connection between the threaded rod and the fixture. The threaded rod thus moves closer to the sensor and finally applies pressure on the sensor. In this way, the pressure applied on the sensor can be adjusted by controlling the rotation angle of the knob. The relationship between the rotation angle of the knob and the applied pressure is calibrated, as shown in Figure 8.

## 3. Results

### 3.1. Pressure Measurement at Cryogenic Temperature

**Figure 9:**
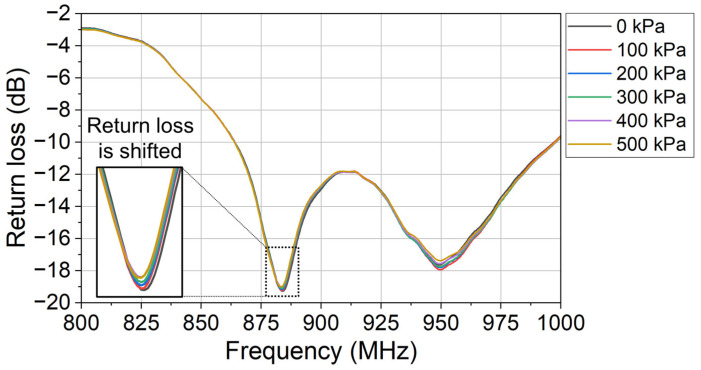
The monitored return loss (raw data) at different applied pressures.

In the experiment, the applied pressure is adjusted by controlling the rotation angle of the knob. At each applied pressure, the corresponding sensor’s return loss (raw data) is recorded wirelessly in the network analyzer, as shown in Figure 9. In Figure 9, it is shown that the sensor’s return loss changes with the applied pressure. The designed sensor successfully modulates the monitored return loss with the applied pressure.

In Figure 9, the monitored return loss includes the contribution of the sensor, the reader antenna, and the surrounding environment [27]. The characteristics of the reader antenna and the surrounding environment are not influenced by the applied pressure on the sensor, which are thus considered as constants in the pressure measurement experiment, as shown in Equations (4) and (5). To extract the contribution of the sensor, the monitored return loss signal (raw data) at 0 kPa of pressure is selected as the reference signal. By calculating the difference between the monitored return loss (raw data) at each applied pressure and the reference return loss, the sensor’s contribution is extracted, as shown in Equation (6).

(4) $$S_{11,raw\_0}=S_{11,sensor\_0}+S_{11,reader}+S_{11,envir}$$

(5) $$S_{11,raw\_p}=S_{11,sensor\_p}+S_{11,reader}+S_{11,envir}$$

(6) $$∆S_{11,raw}=∆S_{11,sensor}=S_{11,raw\_p}-S_{11,raw\_0}$$

In Equations (4)–(6), $S_{11,raw\_0}$ and $S_{11,raw\_p}$ are the monitored return loss (raw data) at 0 kPa of pressure and p kPa of pressure, respectively. $S_{11,sensor\_0}$ and $S_{11,sensor\_p}$ are the sensor’s return loss contribution at 0 kPa of pressure and p kPa of pressure, respectively. $S_{11,reader}$ and $S_{11,envir}$ are the return loss contribution from the reader antenna and the surrounding environment, respectively. $∆S_{11,raw}$ and $∆S_{11,sensor}$ are the variation in the raw data and the sensor’s return loss contribution, compared to the reference signal.

**Figure 10:**
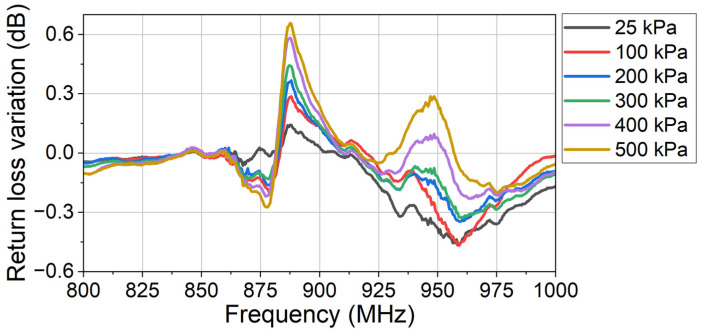
The return loss variation at different applied pressures compared to the reference return loss. In the experiment, the sensor works at cryogenic temperature (−196 °C).

Due to the sensor’s modulation, the variation in return loss at each applied pressure is shown in Figure 10.

In Figure 10, three series of peaks are observed at 877.0 MHz (Peaks 1), 887.0 MHz (Peaks 2), and 948.5 MHz (Peaks 3), respectively. As the applied pressure increases, the return loss variations (absolute value) at 877.0 MHz and 887.0 MHz both increase correspondingly, while the return loss variation at 948.5 MHz does not have a monotonous relationship with the applied pressure. This is because the center frequency of the sensor’s antenna is near 887.0 MHz. Peaks 1 and Peaks 2 are induced by the sensor’s modulation, while Peaks 3 are caused by the evaporation of the liquid nitrogen. Since the dewar is not airtight, the liquid nitrogen evaporates due to the temperature difference between the air (24 °C) and the liquid nitrogen (−196 °C). The evaporated gas nitrogen liquifies the water vapor above the dewar, which is an influence on the backscattering system.

**Figure 11:**
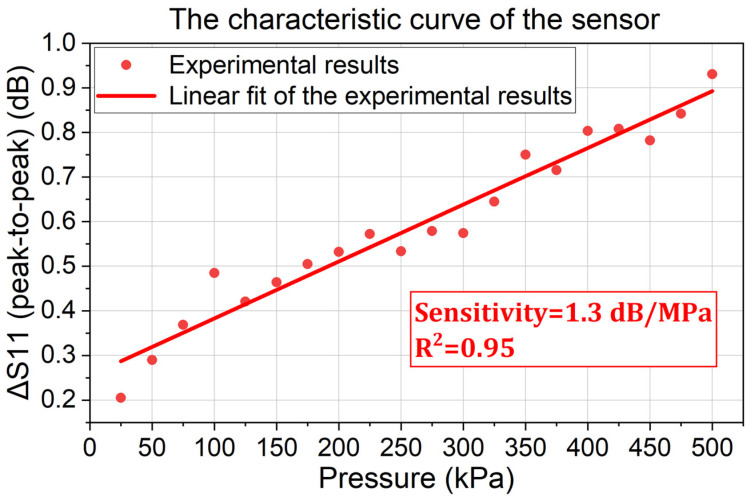
The performance of the proposed pressure sensor at cryogenic temperature (−196 °C).

Since Peaks 1 and Peaks 2 both indicate the sensor’s output signal, the peak-to-peak value from 870.0 MHz to 890.0 MHz is used to present the sensor’s output. The relationship between this peak-to-peak return loss variation (the output) and the applied pressure (the input) is the characteristic curve of the sensor, as shown in Figure 11.

In Figure 11, it is shown that the output of the sensor has a linear relationship with the applied pressure. As the applied pressure increases from 25 kPa to 500 kPa, the peak-to-peak return loss variation increases from 0.21 dB to 0.93 dB. When the sensor works passively and wirelessly in a cryogenic environment (−196 °C), the average sensitivity (i.e., the slope of the characteristic curve) reaches approximately 1.3 dB/MPa. Since the resolution of a typical network analyzer is 0.001 dB, the sensor’s maximum potential resolution can achieve 0.0008 MPa, indicating a high aptitude of the proposed pressure-sensing method in cryogenic applications.

### 3.2. Pressure Measurement at Room Temperature

**Figure 12:**
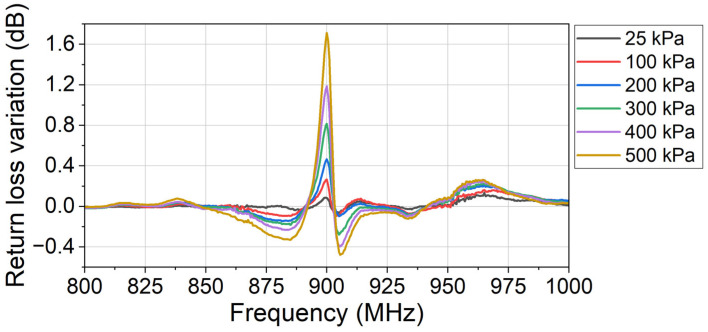
The return loss variation at different applied pressures compared to the reference return loss. In the experiment, the sensor works at room temperature (24 °C).

**Figure 13:**
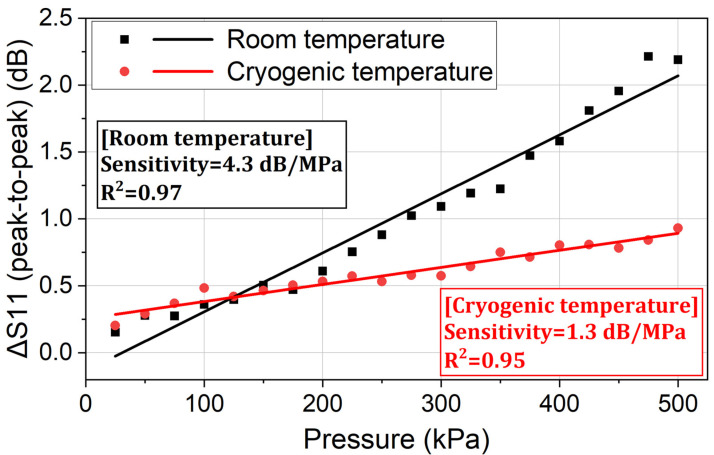
The performance of the proposed pressure sensor at cryogenic temperature (−196 °C) and room temperature (24 °C).

To prove that the designed sensor is functional in a wide range of temperatures, the pressure measurement experiment is also conducted at room temperature. In the room-temperature experiment, no liquid nitrogen was added to the dewar. Using the same test bench and data processing method as in Section 3.1, the return loss variation at each applied pressure is shown in Figure 12. And the sensor’s characteristic at room temperature is shown in Figure 13.

In Figure 12, three series of peaks are observed at 884.0 MHz (Peaks 1), 900.0 MHz (Peaks 2), and 905.5 MHz (Peaks 3), respectively. As the applied pressure increases, the return loss variations (absolute value) at all three peaks increase correspondingly. No peak is observed at around 950 MHz because the liquid nitrogen is not used in the room-temperature experiment. The peak at around 950 MHz in Figure 10 is a spurious peak because of the evaporation of the liquid nitrogen. In Figure 10, only two pressure-sensitive peaks are observed because the third pressure-sensitive peak is submerged in the spurious peak. In the cryogenic experiment, both the sensor and the water vapor modulate the monitored return loss signal. At the submerged third peak, the frequency is not close to the sensor’s center frequency. The sensor’s modulation is not dominant, so the third peak is submerged into the spurious peak.

In Figure 13, it is shown that the output of the sensor has a linear relationship with the applied pressure. As the applied pressure increases from 25 kPa to 500 kPa, the peak-to-peak return loss variation increases from 0.15 dB to 2.19 dB. The average sensitivity reaches 4.3 dB/MPa when the sensor works passively and wirelessly at room temperature. The sensor’s maximum potential resolution can achieve 0.0002 MPa, indicating that the proposed pressure-sensing method is also functional at room temperature.

Figure 13 also shows that the sensor has higher sensitivity at room temperature, compared to the result at cryogenic temperature. This is because the Young’s modulus and the flexural modulus of the sensor’s package increase at cryogenic temperature [28]. Higher pressure is needed to achieve the same deformation at cryogenic temperature. Furthermore, at a temperature of 77 K, it is reported that the remanent field of an NdFeB N35 magnet is just 93% of the one at 300 K [29]. Additionally, the sensitivity of the TMR at 77 K is 93.4% of the one at 300 K. According to the theoretical model in Section 2.2, the TMR’s resistance variation at cryogenic temperature is smaller than the variation at room temperature. Thus, the return loss variation of the sensor is smaller, and the sensitivity is lower at cryogenic temperatures.

Figure 10 and Figure 12 also show that the sensor’s center frequency is stable during the measurement. The maximum frequency shift is 1.0 MHz at cryogenic temperature (from 887.0 MHz to 888.0 MHz) and 0.5 MHz at room temperature (from 899.5 MHz to 900.0 MHz). This is important to achieve a high accuracy for the presented sensor. In the backscattering system, the frequency response of the reader antenna is nonlinear. The transmitted signal has different power at different frequencies. If the sensor’s center frequency shifts notably, the sensor will receive more (or less) power. As a result, the sensor’s backscattered power is different from the calibrated one, which will impact the accuracy of the sensor.

In summary, the proposed wireless, passive pressure-sensing method and the designed sensor are proved to be functional for a wide range of temperatures, from cryogenic temperatures (−196 °C) to room temperature (24 °C). Due to the sensor’s simple structure and wireless, passive, low-cost characteristics, the proposed method is believed to be desirable for cryogenic applications and future Internet of Things (IoT) applications.

## 4. Discussion

### 4.1. Multisensor Measurement

In cryogenic applications such as gas recovery, a large sensor array is used to monitor the pressure at different positions. To simplify the monitoring system, it is desirable that all sensors can be wirelessly and simultaneously read by a single reading device. In this scenario, the main challenge is to decouple the monitored return loss signal to extract the output signal of each sensor.

This challenge can be overcome by separating the working frequency band of each sensor. In Figure 10 and Figure 12, it is shown that the designed sensor only modulates a narrow frequency band of the monitored return loss signal. By tuning the sensor’s center frequency, the sensors can work in separated frequency bands and thus be independent of each other. To prove the feasibility of such a multisensor measurement scheme, two sensors are fabricated for simultaneous pressure measurement. The two sensors use different capacitors in the load circuit (Figure 5) to achieve different center frequencies. In Sensor 1, the TMR is in series connection with a 1.0 pF capacitor. In Sensor 2, the TMR is in series connection with a 2.7 pF capacitor (Sensor 2 is the sensor evaluated in Chapter 3). Other parts of the two sensors are identical. The test bench is the same as the one shown in Figure 7, and the two sensors are placed in two different dewars.

**Figure 14:**
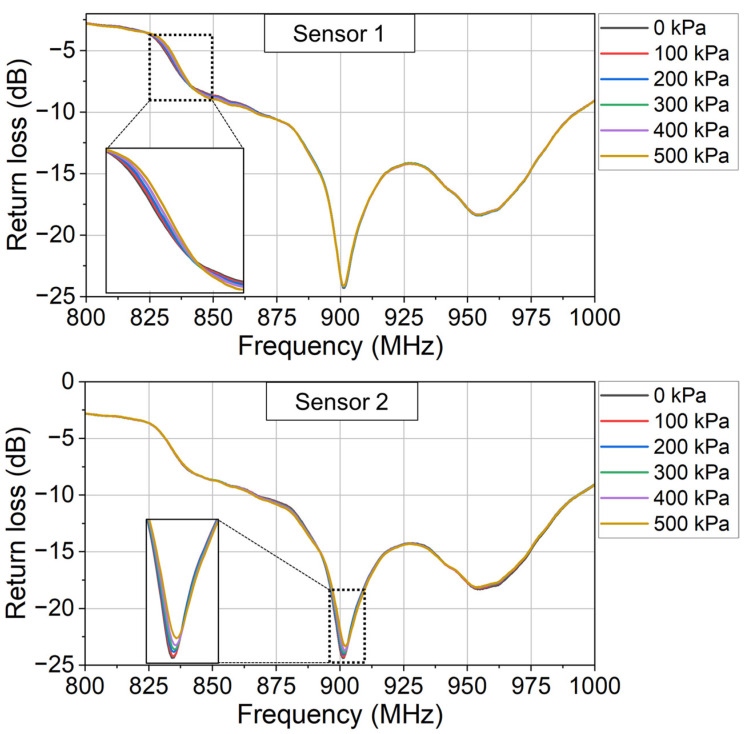
The monitored return loss (raw data) at different applied pressures. This is the first step to calibrate the fabricated two sensors.

**Figure 15:**
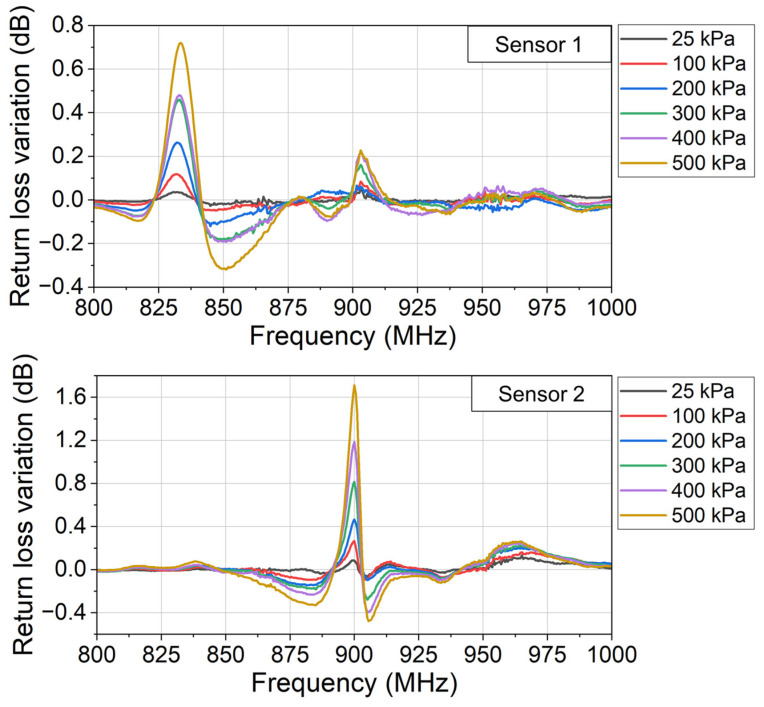
The return loss variation due to the sensor’s modulation. This is the second step to calibrate the fabricated two sensors. Sensor 2 is the sensor which is evaluated in Chapter 3.

**Figure 16:**
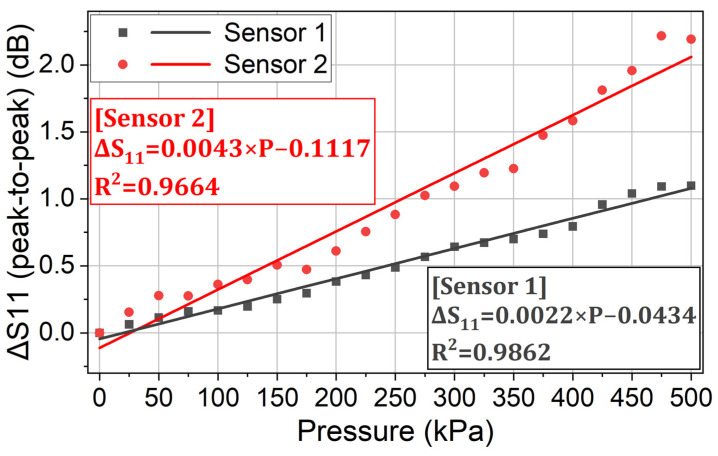
The characteristic curve of each sensor. This is the last step to calibrate the fabricated two sensors. The calibrated model will be used in the multisensor measurement to decouple the monitored output signal.

In the experiment, the characteristic curves of the two sensors are firstly calibrated. The monitored return loss (raw data) at different applied pressures is shown in Figure 14. Using the same data processing method as in Section 3.1 (Equations (4)–(6)), the return loss variation due to each sensor’s modulation is derived as Figure 15. And the characteristic curve of each sensor is derived as Figure 16.

In Figure 16, both sensors show high linearity and sensitivity in the pressure measurement. Sensor 1 has a lower sensitivity (2.2 dB/MPa) than Sensor 2 (4.3 dB/MPa) due to the frequency characteristics of the reader antenna. As shown in Figure 14, the monitored return loss at 833 MHz is lower than the one at 900 MHz. This is because the used reader antenna is not a wide-band antenna. The reader antenna has lower performance and transmitted power at 833 MHz, compared to the parameters at 900 MHz. As a result, the backscattered power from Sensor 1 is lower than the backscattered power from Sensor 2. Sensor 1 modulates a smaller part of the monitored return loss and thus has a lower sensitivity than Sensor 2.

**Figure 17:**
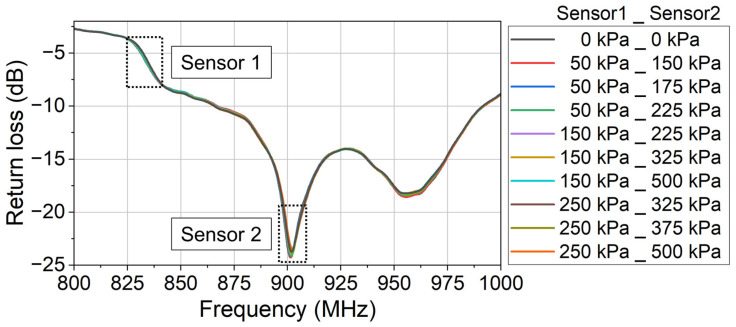
The monitored return loss (raw data) at different applied pressures. Different pressures are simultaneously applied on the two sensors. The output signals of the two sensors are included in the different frequency bands of the monitored return loss signal.

After calibrating the two sensors, the multisensor measurement experiment is conducted. Random pressure is applied simultaneously on the two sensors. For each group of applied pressures, the monitored return loss is shown in Figure 17.

**Figure 18:**
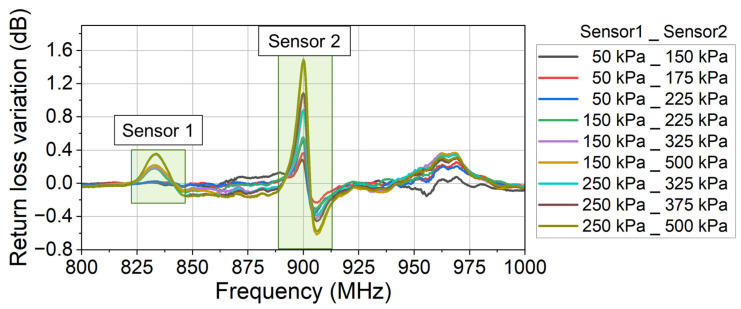
The return loss variation due to the sensors’ modulation. Different pressures are simultaneously applied on the two sensors. The two sensors modulate different frequency bands of the monitored return loss signal and are thus independent of each other.

In Figure 17, the legend in the format “a_b” means that the applied pressure on Sensor 1 is a kPa and the applied pressure on Sensor 2 is b kPa. The reference return loss signal is measured when no pressure is applied on either of the two sensors. Using the same data processing method shown in Section 3.1, the return loss variation due to the sensors’ modulation is derived as Figure 18.

**Figure 19:**
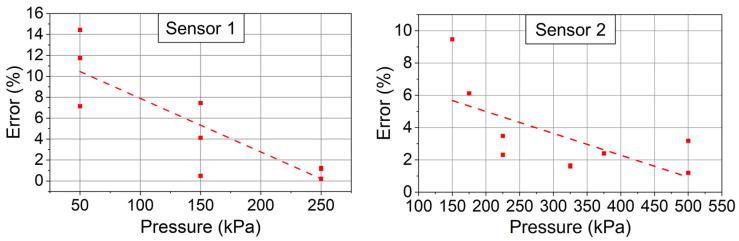
The error of the fabricated two pressure sensors in the multisensor measurement. The dotted lines are the fitted curves of the measurement error.

In Figure 18, the return loss variation is observed in two different frequency bands, corresponding to the signals from Sensor 1 and Sensor 2, respectively. Using the return loss variation in Figure 18 and the calibrated model in Figure 16, the measured pressure is derived as Table 3. The measurement error is calculated, as shown in Table 3 and Figure 19.

In Figure 19, it is shown that the measurement error of Sensor 1 is lower than 15% and the measurement error of Sensor 2 is lower than 10%. The measurement error decreases with the applied pressure. This is because the sensor-induced return loss variation is larger when higher pressure is applied. The percentage of the noise-induced return loss variation is smaller, so the measurement error is lower at higher pressure.

In Figure 19, the measurement error is induced due to three main reasons: First, the surrounding environment continuously changes during the measurement. Changes in the surrounding environment (e.g., the motion of surrounding people) bring interference to the whole backscattering system, which leads to a measurement error. Second, the signal-to-noise ratio (SNR) of the backscattering system is not enough. Since the maximum output power of the network analyzer is only 5 dBm (about 3 mW), the noise is unneglectable. Third, the liquid nitrogen evaporates during monitoring. The evaporated liquid nitrogen is cold and the water vapor in the air turns into liquid water, which also modulates the backscattered signal.

There are four main ways to reduce measurement errors. First, the antenna design and the load design can be optimized to achieve higher antenna gain and better impedance matching between the antenna and the load. In this way, the sensor’s sensitivity can be further enhanced, and the sensor’s modulation can be more dominant in the backscattering system. As a result, the changes in the surrounding environment will be less dominant and bring smaller errors. Second, an RF signal generator can be used to enhance the power of the transmitted signal. An RF circulator should be used at the same time to separate the transmitted signal and the backscattered signal. The enhancement of the transmitted power will improve the system’s SNR. Third, the evaluation system can be optimized to decrease the heat transfer between the liquid nitrogen and the air. Last, the sensor’s sensitivity can be further enhanced by changing the material or the structure of the sensor’s package, which will be detailed in the next section.

In real measurements, the microwave measurement system should be correctly installed and calibrated. First, the reader antenna should be installed on a stable supporter without slippage. The reader antenna is then connected to the network analyzer by an RF cable. Second, the length of the RF cable should be calibrated in the network analyzer. Third, the sensors should be installed at their working positions. Fourth, the sensors and the surrounding environment should be calibrated. At least three different pressures should be applied to the sensors, and the corresponding monitored return loss will be used for calibration. After finishing the above preparations, the sensor’s sensitivity, linearity, and precision are expected to reach the experimental value.

In summary, multisensor measurement is successfully achieved using a single reading device. By separating the frequency band of each sensor, the monitored return loss signal can be easily decoupled and the signal from each sensor can be extracted. Using the return loss variation signal induced by the sensor array and the calibrated characteristic curve of each sensor, the pressure applied on each sensor can be obtained.

### 4.2. Sensitivity and Measurement Range

In the experiment, it is found that the sensor’s return loss variation is linear to the applied pressure and the relative displacement between the magnet and the TMR. By changing the structure or material of the sensor’s package, the relative displacement can be more sensitive to the applied pressure. The sensor’s return loss variation can become larger at the same applied pressure, and the sensor’s sensitivity can be further enhanced.

Similarly, the sensor’s measurement range can also be extended. Currently, the measurement range of the sensor is physically limited by the initial distance between the MR and the magnet. When the MR contacts the magnet due to pressure-induced deformation, the corresponding applied pressure is the maximum measurable pressure. By revising the structure and material of the sensor’s package, the relative displacement can be less sensitive to the applied pressure. Larger pressure is needed to achieve the maximum relative displacement, and the sensor’s measurement range can be further extended.

COMSOL simulations are conducted to prove the above discussions. In the simulation, a constant pressure (100 kPa) is applied on the sensor. In the simulation, the thickness and the Young’s modulus of the deformed part are revised. For each revised sensor, the corresponding relative displacement between the magnet and the TMR ($∆d_{n}$) is simulated. As a comparison, the old sensor (the sensor used in the experiment) is also simulated in the same conditions. For the old sensor, the corresponding relative displacement between the magnet and the TMR is $∆d_{o}$. Based on the above analysis, the ratio $∆d_{n}/∆d_{o}$ shows the sensitivity ratio of a revised sensor to the old sensor (${Sens}^{\prime }/Sens$). Here, Sens is the sensitivity of the old sensor, which is measured in the experiment. ${Sens}^{\prime }$ is the expected sensitivity of a revised sensor, which can be calculated using the simulation results and Equation (7).

(7) $${Sens}^{\prime }=\left(∆d_{n}/∆d_{o}\right)\times Sens$$

Similarly, the ratio $∆d_{o}/∆d_{n}$ shows the measurement range ratio of a revised sensor to the old sensor (${Mea}^{\prime }/Mea$). Here, Mea is the measurement range of the old sensor, which is obtained in the experiment. ${Mea}^{\prime }$ is the expected measurement range of a revised sensor, which can be calculated using the simulation results and Equation (8).

(8) $${Mea}^{\prime }=\left(∆d_{o}/∆d_{n}\right)\times Mea\;$$

**Figure 20:**
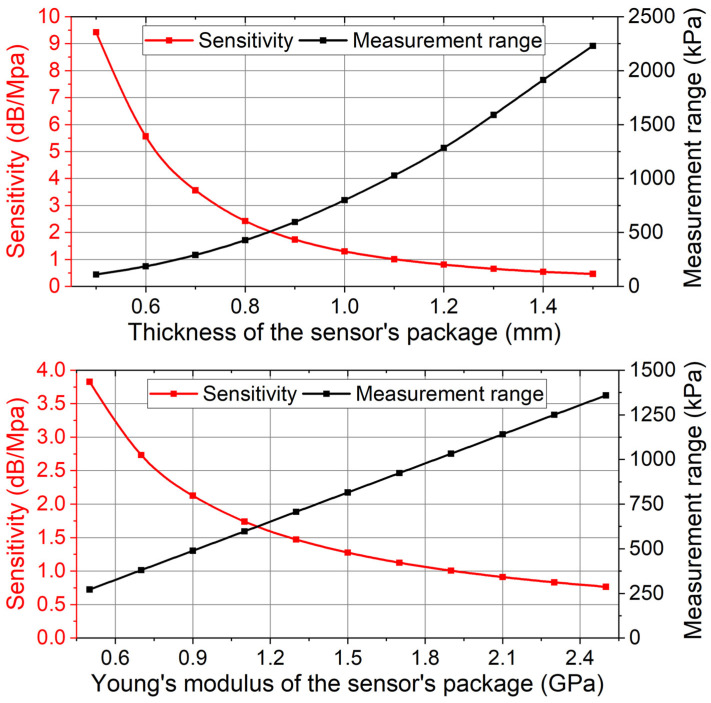
Expected sensitivity and measurement range of the sensor after revising the structure and the material of the sensor’s package.

Based on the COMSOL simulation results ($∆d_{n},∆d_{o}$), the experimental results (Sens,Mea), and Equations (7) and (8), the sensor’s sensitivity and measurement range are related to the thickness and Young’s modulus of the sensor’s package, as shown in Figure 20.

Figure 20 shows that the sensitivity is expected to be enhanced to 9.4 dB/MPa by simply reducing the thickness of the deformed part. The measurement range is expected to be extended to 2229 kPa by simply increasing the thickness of the deformed part. Similarly, the sensor’s sensitivity and measurement range can also be adjusted by changing the material of the sensor’s package. The sensitivity is expected to be enhanced to 3.8 dB/MPa by using a material with a Young’s modulus of 0.5 GPa. The measurement range is expected to be extended to 1359 kPa by using a material with a Young’s modulus of 2.5 GPa.

### 4.3. The Effectiveness of the Presented Sensing Principle to Different Applications

As mentioned in the introduction and verified in the pressure measurement, an MR-integrated backscattering antenna can perform both vibration sensing and pressure sensing. The issue of whether the sensing principle is more effective for vibration sensing or pressure sensing depends on the sensor’s working condition.

In an unstable environment with diverse interferences, the sensing principle is more effective for vibration sensing. A change in the sensor’s surrounding environment applies an offset on the calibrated “zero point” (the monitored return loss when no vibration or pressure is applied). However, the derivation of vibration does not use the calibrated “zero point”. Instead, the vibration is derived from the peak-to-peak value of a time-domain return loss signal. The peak-to-peak value mainly depends on the sensor’s modulation effect and is not obviously influenced by environmental changes. On the contrary, the derivation of pressure depends on how much the return loss is shifted from the calibrated “zero point”. Environmental changes have an impact on the accuracy of pressure sensing. Thus, vibration sensing is more robust than pressure sensing in unstable environments.

In a stable environment, the sensing principle is more effective for pressure sensing. In the monitoring of a normal piece of equipment, the vibration-induced cantilever displacement is generally less than 1 mm. Although the cantilever displacement can be further enhanced by designing a longer and thinner cantilever, this will negatively influence the sensor’s lifespan. On the contrary, the pressure-induced deformation can achieve 2 mm or higher in pressure measurement. As a result, for pressure sensing, it is easier to achieve higher sensitivity in the real measurement.

### 4.4. Future Work

For sensors working in cryogenic conditions, the sensor’s sustainability is an important issue. Long-term continuous monitoring is needed to obtain a convincing and comprehensive dataset. In future work, the repeatability, long-term life, and reliability of the sensor will be evaluated to prove the sensor’s sustainability.

## 5. Conclusions

In this paper, we developed a novel pressure-sensing method via an MR-integrated antenna and a magnet. Our method is functional at cryogenic temperatures because all employed components are low-temperature-tolerant passive devices. Our method achieves wireless passive measurements because the monitored pressure is passively transferred to a wirelessly obtainable return loss. A sensor prototype was developed and evaluated at room temperature (24 °C) and cryogenic temperature (−196 °C) to verify our method. The sensitivity reached 4.3 dB/MPa at 24 °C and 1.3 dB/MPa at −196 °C, demonstrating a high aptitude for wireless passive pressure measurement in a wide temperature range. The frequency band of the sensors can be easily tuned by changing the connected capacitor. Simultaneous measurements with a sensor array were therefore achieved via a single reading device. Our method performs low-cost, simple, robust, passive, and wireless pressure measurement at −196 °C; thus, it is desirable for cryogenic applications.

## Figures and Tables

**Table 1:** The dimension of the designed antenna.

Parameter	Value (mm)	Parameter	Value (mm)
La	43.4	Wa	19.0
Lr	4.0	W1	9.2
Lw	0.8	W2	15.2
Ls	0.8	L1	5.0
Lf	2.0	L2	10.0

**Table 2:** Dimension, material, and cost of each component in the sensor.

Component	Dimension (mm)	Material/Type	Cost (USD)
Antenna	43.4 × 19.0 × 0.16	Copper	0.47
TMR	2.95 × 1.65 × 1.45	TMR2104LS	1.26
Magnet	3.4 × 3.4 × 3.0	NdFeB	0.13
Lid	57.0 × 30.0 × 2.0	PETG	0.14
Substrate	57.0 × 30.0 × 8.0	PETG	0.38

**Table 3:** The measurement error of the fabricated two pressure sensors.

Number	Sensor 1			Sensor 2		
	Real (kPa)	Measured (kPa)	Error	Real (kPa)	Measured (kPa)	Error
1	50.00	42.78	−14.44%	150.00	164.21	9.47%
2	50.00	44.13	−11.74%	175.00	164.29	−6.12%
3	50.00	46.43	−7.14%	225.00	230.22	2.32%
4	150.00	150.74	0.49%	225.00	232.84	3.49%
5	150.00	143.81	−4.13%	325.00	330.18	1.59%
6	150.00	161.17	7.45%	500.00	515.91	3.18%
7	250.00	247.13	−1.15%	325.00	319.62	−1.66%
8	250.00	249.48	−0.21%	375.00	384.02	2.41%
9	250.00	253.12	1.25%	500.00	506.01	1.20%

## Data Availability

The data presented in this study are available on request from the corresponding author.
